# Substantially more children receiving antidepressants see a specialist than reported by Jack et al.

**DOI:** 10.1186/s12916-023-03043-x

**Published:** 2023-09-11

**Authors:** Vicky P. Taxiarchi, Carolyn A. Chew-Graham, Matthias Pierce

**Affiliations:** 1 Division of Psychology and Mental Health, Faculty of Biology, Medicine and Health, Centre for Women’s Mental Health, University of Manchester, Jean McFarlane Building, Oxford Road, Manchester, M13 9PL UK; 2 School of Medicine, Keele University, Keele, Staffordshire UK

**Keywords:** Children and young people (CYP), Antidepressants, Prescriptions, Primary care, Secondary care

## Abstract

We would like to draw attention to evidence of substantial bias in the article published in this journal by Jack et al. (BMC Med 18:1-12, 2020). They provide an analysis of antidepressant prescribing to children and young people (CYP; ages 5 to 17) in primary care in England and reported that only 24.7% of CYP prescribed SSRIs for the first time were seen by a child and adolescent psychiatrist—contrary to national guidelines. We believe that their analysis is based on incomplete data that misses a large proportion of specialist mental health contacts. This is because the dataset Jack et al. used to capture specialist mental health contact—The Hospital Episode Statistics (HES) dataset—has poor coverage, as most CYP mental health services do not submit data. We demonstrate the level of underreporting with an analysis of events in a large primary care dataset where there has been a record of definite contact with CYP mental health services. We report that as many as three quarters of specialist CYP contacts with mental health specialists are missed in the HES dataset, indicating that the figure presented by Jack et al. is substantially wrong.

## Methods

In order to quantify the level of underreporting of CYP mental health services report to the HES dataset, we use the Clinical Research Practice Datalink (CPRD-Aurum) linked to HES inpatient and outpatient datasets. The CPRD records diagnoses, medications and other clinical events, including referrals and discharges from hospital. We identified 4,046,885 CYP, aged 5 to 17, who were eligible for linkage to HES and registered at a CPRD-Aurum participating practice over the period 1 January 2007 to 31 December 2017. We selected observations in the CPRD which have been coded as “Seen in child and adolescent psychiatry clinic” or “Seen by child and adolescent psychiatrist”. We included only those events occurring over the age range 5 to 17 and between 2007 and 2017. When children had more than one event, the first was selected. This resulted in a cohort of 15,726 CYP. We then calculated the proportion of these who had an inpatient or outpatient HES records in the year before this event, where the clinical speciality was “Child and Adolescent Psychiatry”. This was our main measure, which we interpret as the proportion of CYP mental health specialists that report to HES.

As a sensitivity analysis, we expanded the capture window to 2 years before and included other clinical specialties: “Adult Mental Illness”, “Forensic Psychiatry”, “Paediatric Neurology”, “Paediatrics”, “Medical Psychotherapy”, “Neurology”. These clinical specialisms emulate other specialisms included in the analysis by Jack et al. [1]. As a further sensitivity, we restricted to events that occurred in the CPRD from 1 April 2009 onwards as data quality in the HES outpatient dataset has been reported to be better from April 2008 [2].

## Results

Of 15,726 CYP with a contact with a CYP psychiatrist, only 27.5% (*n* = 4325) were matched to a HES record in the year before, where the clinical speciality was ‘Child and Adolescent Psychiatry’ (Table 1). Following expansion to other specialists, the rate of matches increased to 49.0%. By widening the data capture window to 2 years prior, the rates of matches increased to 29.8% and 56.0%.

**Table 1:** Rates of matches between CPRD records where there was a record of contact with child and adolescent psychiatrist, and HES records [95% CI]

		Specialists	
		Child psychiatrist	Child psychiatrist and others
Capture window	1 year	27.5 [26.8, 28.2]	49.0 [48.3, 49.8]
	2 years	29.8 [29.1, 30.5]	56.0 [55.2, 56.8]

*CI* Confidence interval

## Discussion

We report that, in England, between three quarters and a half of contacts with specialist CYP mental health services are not reported in national hospital data. We conclude, therefore, that the paper by Jack et al [1]. considerably underestimates the true rate of contacts with specialist CYP mental health services for children and adolescents who are prescribed antidepressants, and the implication that general practitioners (GPs) are initiating antidepressants in children and young people in the majority of cases is simply wrong.

Whilst some CYP may be prescribed antidepressants against clinical guidelines, the figure of only 25% seeing a specialist is inaccurate and unnecessarily alarmist. From our analysis, if we apply the figure of 27.5% of CYP mental health services being in the HES dataset, then we could estimate that the figure is closer to $25/0.275$ = 91%, which fits more closely to our clinical experience. Given that we use a different primary care dataset to Jack et al., further checks are needed to ascertain that our estimate applies to their sample. Otherwise, it may be possible to re-do their analysis, using a sample of primary care practices where we know that data are being reported to HES. Whilst this task may be challenging given the quality of the data at hand, getting the true figure correct is important, as the findings have significant public interest, demonstrated by the results by Jack et al. being repeated in a report by the National Institute for Health and Care Research [3] and subsequently picked up in the national media [4, 5]. Having inaccurate statistics serves nobody and is likely to cause unwarranted alarm amongst the public and policymakers and confusion and upset amongst GPs who do follow guidelines.

Our analysis also demonstrates a significant data gap. Whilst data are not routinely reported from specialist CYP mental health services to the HES dataset, they are reported to a separate national dataset, the Mental Health Services Dataset (MHSDS) [6]. However, there are significant data quality issues with this dataset, and, because of this, linkage between this and the CPRD has been withdrawn. Whilst analysis of the MHSDS has helped show trends in referrals to specialist mental health services, the poor quality of recording of patient characteristics and lack of linkage to other datasets severely limits its utility [6]. In particular, there is a gap in our understanding of who is being seen in services and what their long-term outcomes are. Better quality data and linkage is vital if we are to tackle significant public health challenges, such as the current crisis in CYP mental health treatment.

## Data Availability

The clinical codes, data management and analysis code used in this study are available on request from the corresponding author. Access to data can be requested via application to the Clinical Practice Research Datalink.

## Abbreviations

CYP: Children and young people
HES: Hospital Episode Statistics
CPRD: Clinical Research Practice Datalink
GPs: General practitioners
MHSDS: Mental Health Services Dataset
